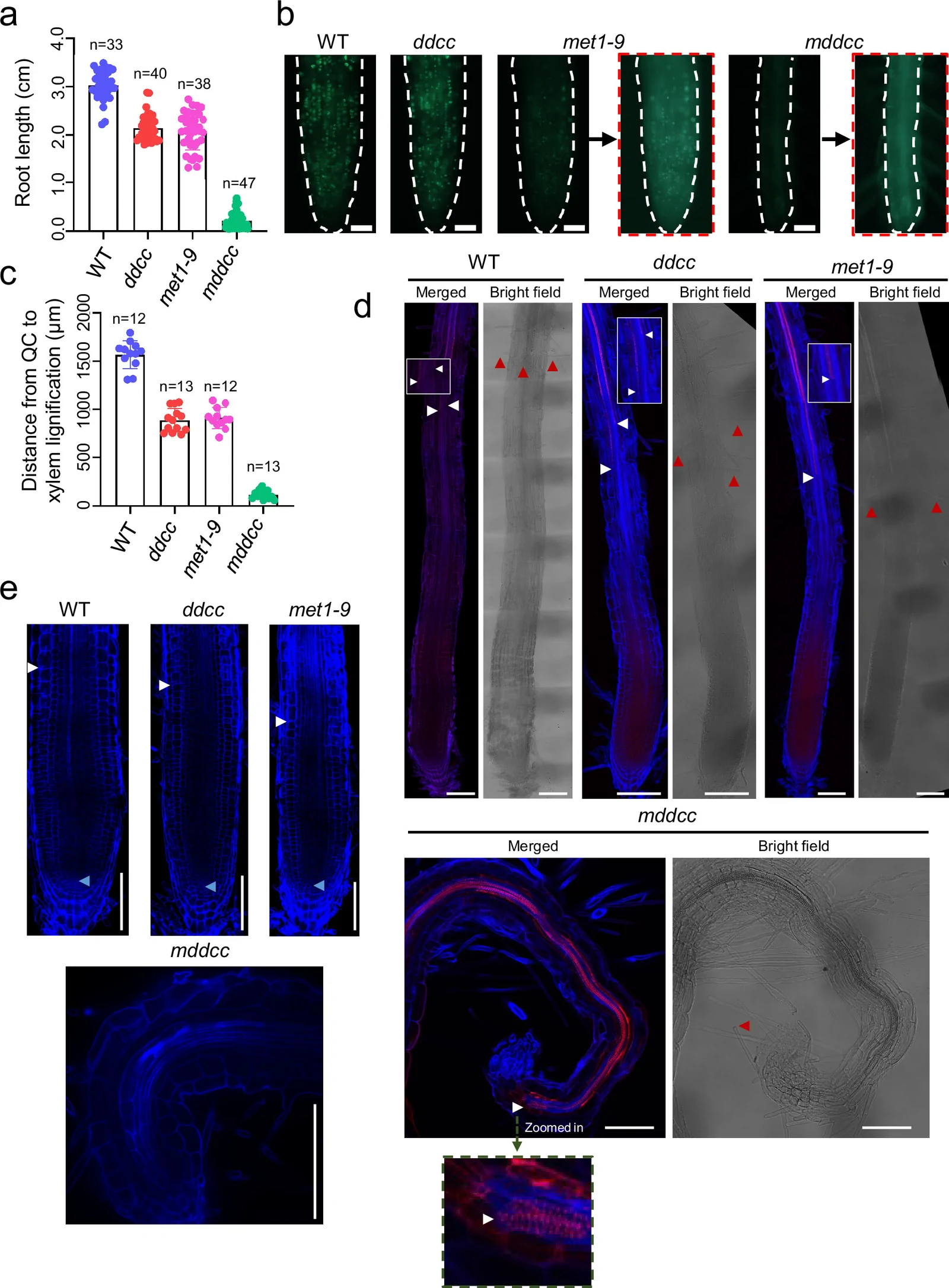# Author Correction: DNA methylation-free *Arabidopsis* reveals crucial roles of DNA methylation in regulating gene expression and development

**DOI:** 10.1038/s41467-026-78327-w

**Published:** 2026-10-02

**Authors:** Li He, Huan Huang, Mariem Bradai, Cheng Zhao, Yin You, Jun Ma, Lun Zhao, Rosa Lozano-Durán, Jian-Kang Zhu

**Affiliations:** 1 Shanghai Center for Plant Stress Biology, Center for Excellence in Molecular Plant Sciences, Chinese Academy of Sciences, 201602 Shanghai, China; 2 Department of Clinical Science Intervention and Technology, Karolinska Institutet, 14186 Stockholm, Sweden; 3 University of the Chinese Academy of Sciences, 100049 Beijing, China; 4 Department of Plant Biochemistry, Centre for Plant Molecular Biology (ZMBP), Eberhard Karls University, D-72076 Tübingen, Germany; 5 Present Address: Institute of Advanced Biotechnology and School of Life Sciences, Southern University of Science and Technology, 518055 Shenzhen, China

Correction to: *Nature Communications* https://doi.org/10.1038/s41467-022-28940-2, published online 14 March 2022

In the version of the article initially published, there were copy–paste errors in the Fig. 6a source data. The Source Data file and figure have now been corrected, as seen in Fig. 1 below. This correction has been made to the HTML and PDF versions of the article.

Fig. 1 Original and corrected Fig. 6a

**Original Fig. 6a**:

**Figure:**
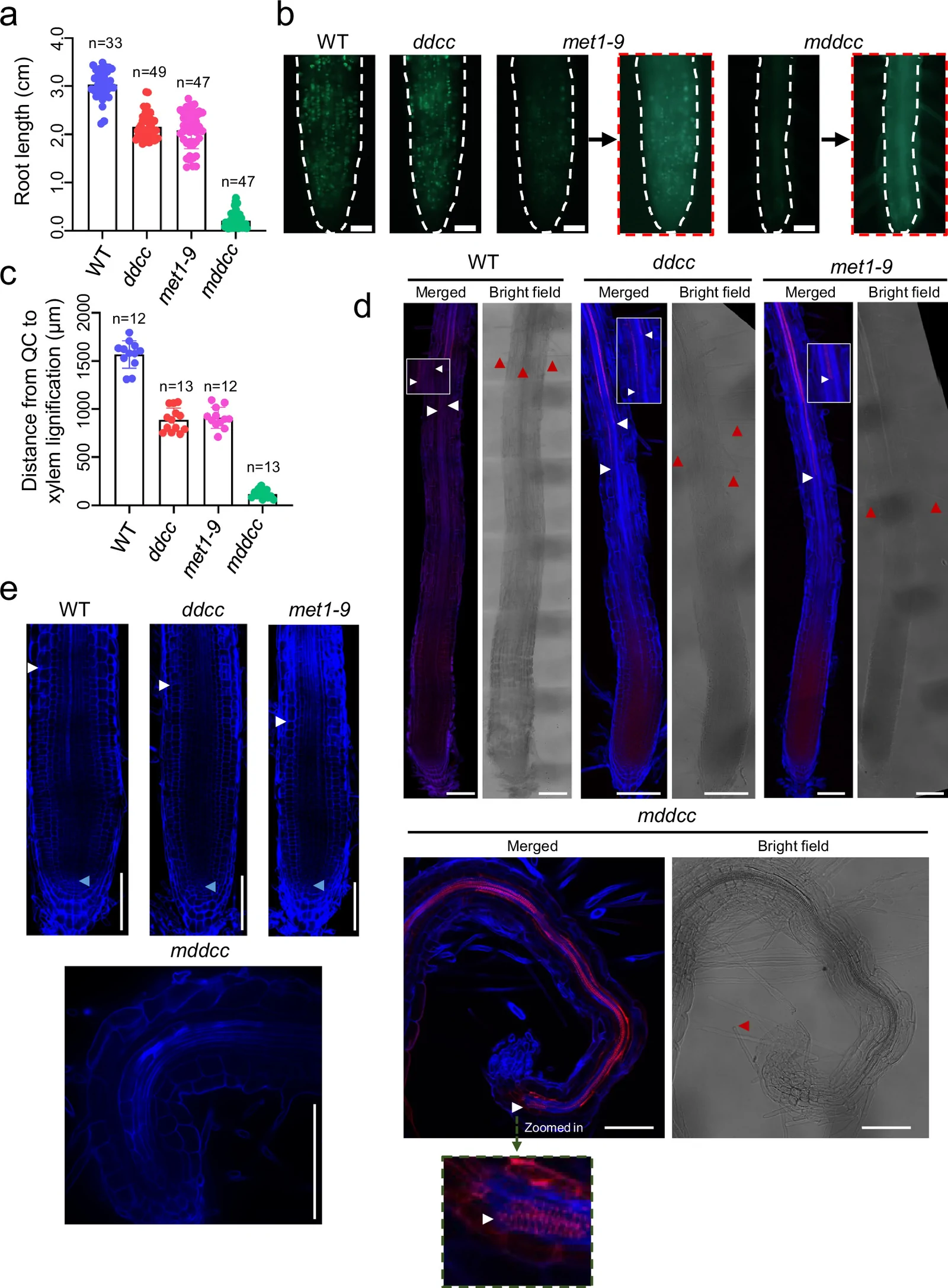

**Corrected Fig. 6a**:

**Figure:**