# Supplementary material for: DNA methylation-free Arabidopsis reveals crucial roles of DNA methylation in regulating gene expression and development
Source: Nat Commun. 2022 Mar 14;13:1335. doi: 10.1038/s41467-022-28940-2 (PMC8921224; doi:10.1038/s41467-022-28940-2)
Supplement: Supplementary file 1 — Supplementary Information [file 41467_2022_28940_MOESM1_ESM.pdf]

DNA methylation-free *Arabidopsis* reveals crucial roles of DNA methylation in regulating gene expression and development

He *et al.*

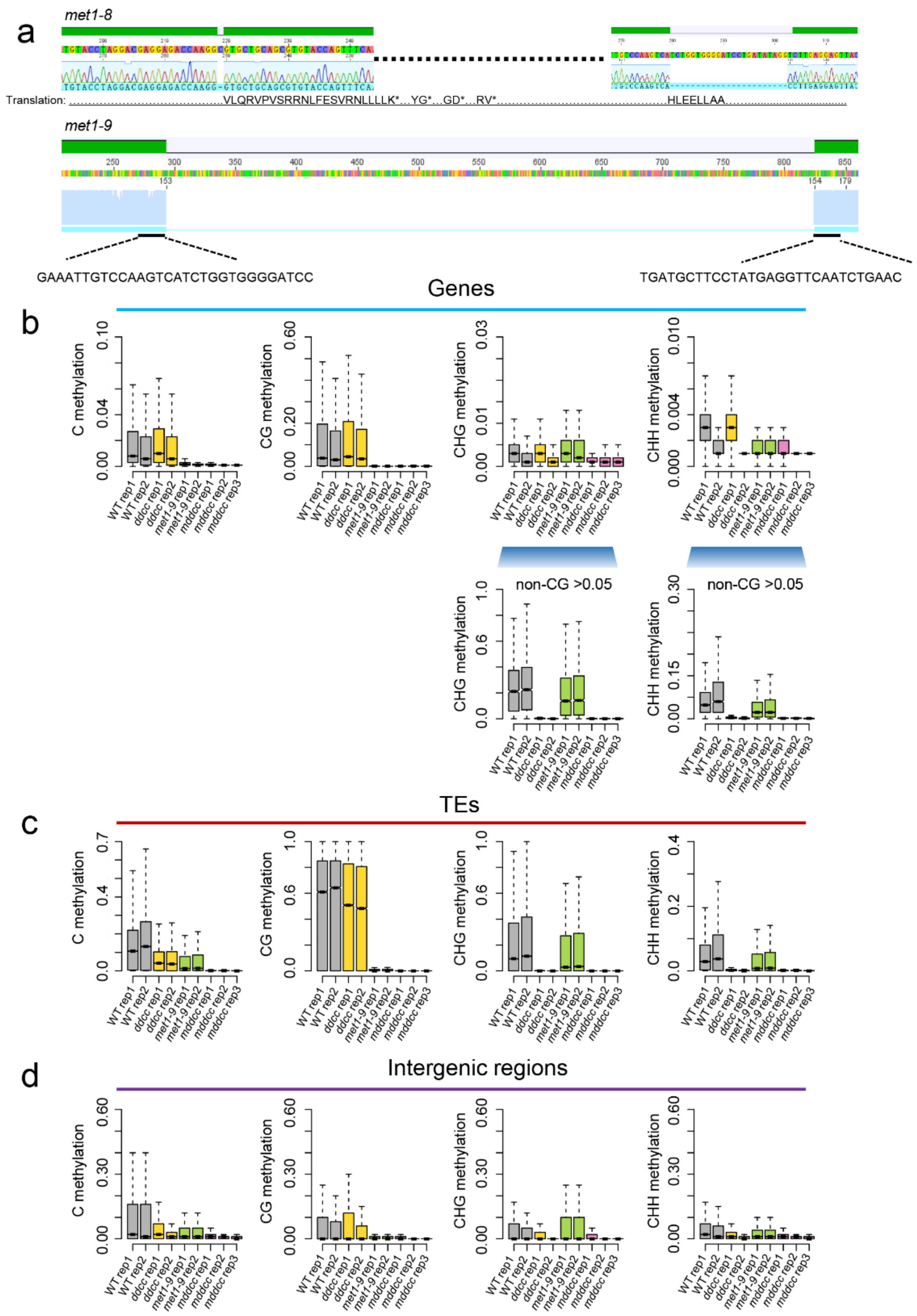

Supplementary Figure 1. Comparison of the DNA methylation levels in WT, *ddcc*, *met1-9*, and *mddcc*. (a) Sequence of *met1-8* and *met1-9* alleles aligned to wild-type *MET1*. The translation track shows that the deletion in *met1-8* results in a premature stop codon. (b)-(d) Box plots showing DNA methylation levels of all genes (b), all TEs (c), and all intergenic regions (d) in the indicated mutants. Since most genes do not carry non-CG methylation, the comparison of CHG and CHH methylation levels in genes with non-CG methylation > 0.05 in the WT is shown in the lower panel (b). The horizontal line within the box represents the median; the whiskers represent minimum and maximum values; and the lower and upper boundaries of the box represent the 25th and 75th percentiles, respectively.

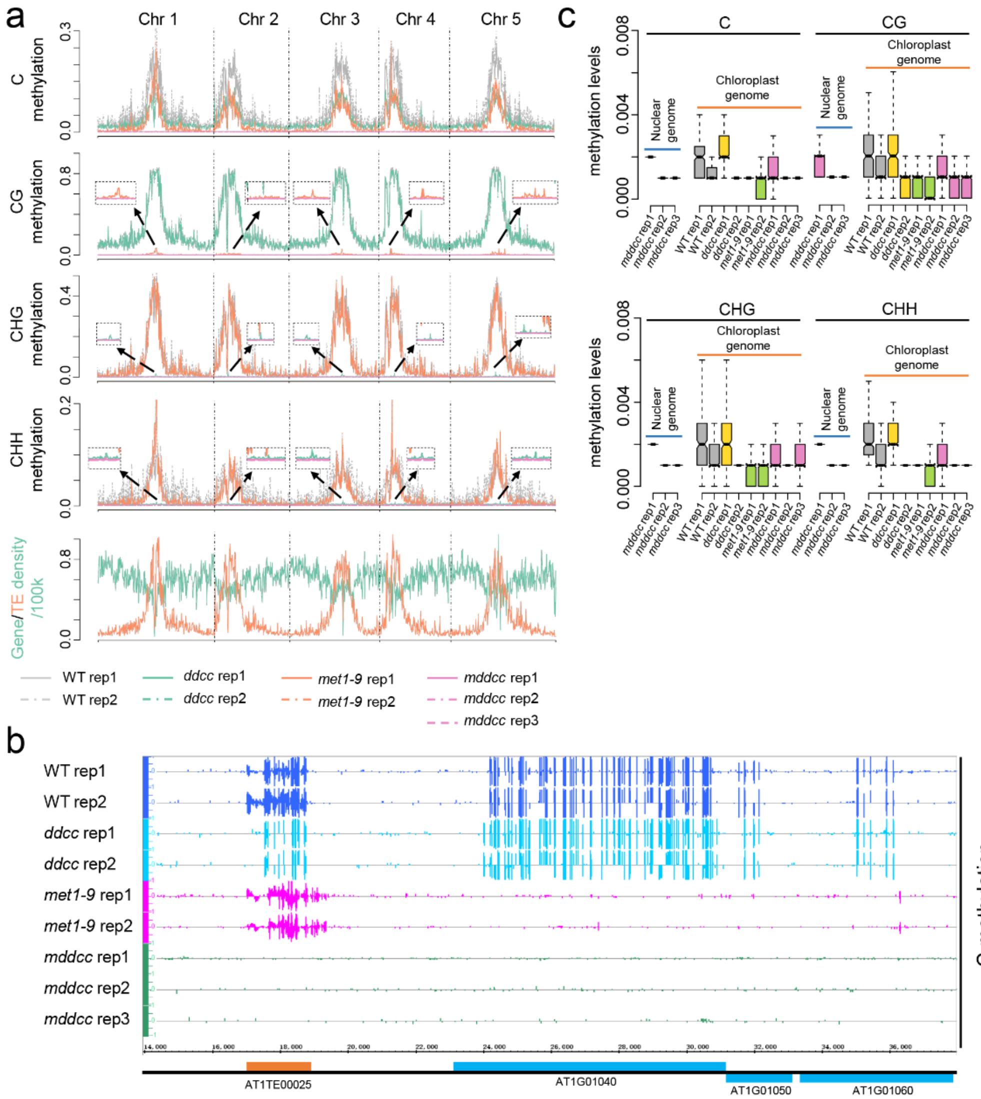

Supplementary Figure 2. DNA methylation is eliminated in the *mddcc* mutant. (a) Genome-wide distribution of DNA methylation in all three sequence contexts in the indicated mutants. Wireframes are zoom-in of the indicated regions. It should be noted that the lines of the WT overlap with those of *ddcc* in the mCG row. (b) Screenshot of DNA methylation levels over one representative locus in the indicated mutants. Orange and blue bars indicate TEs and genes, respectively. TEs and genes oriented 5' to 3' and 3' to 5' are shown above and below the line, respectively. (c) Comparison of the DNA methylation levels between nuclear and chloroplast genomes in the indicated genotypes. The horizontal line within the box represents the median; the whiskers represent minimum and maximum values; and the lower and upper boundaries of the box represent the 25th and 75th percentiles, respectively.

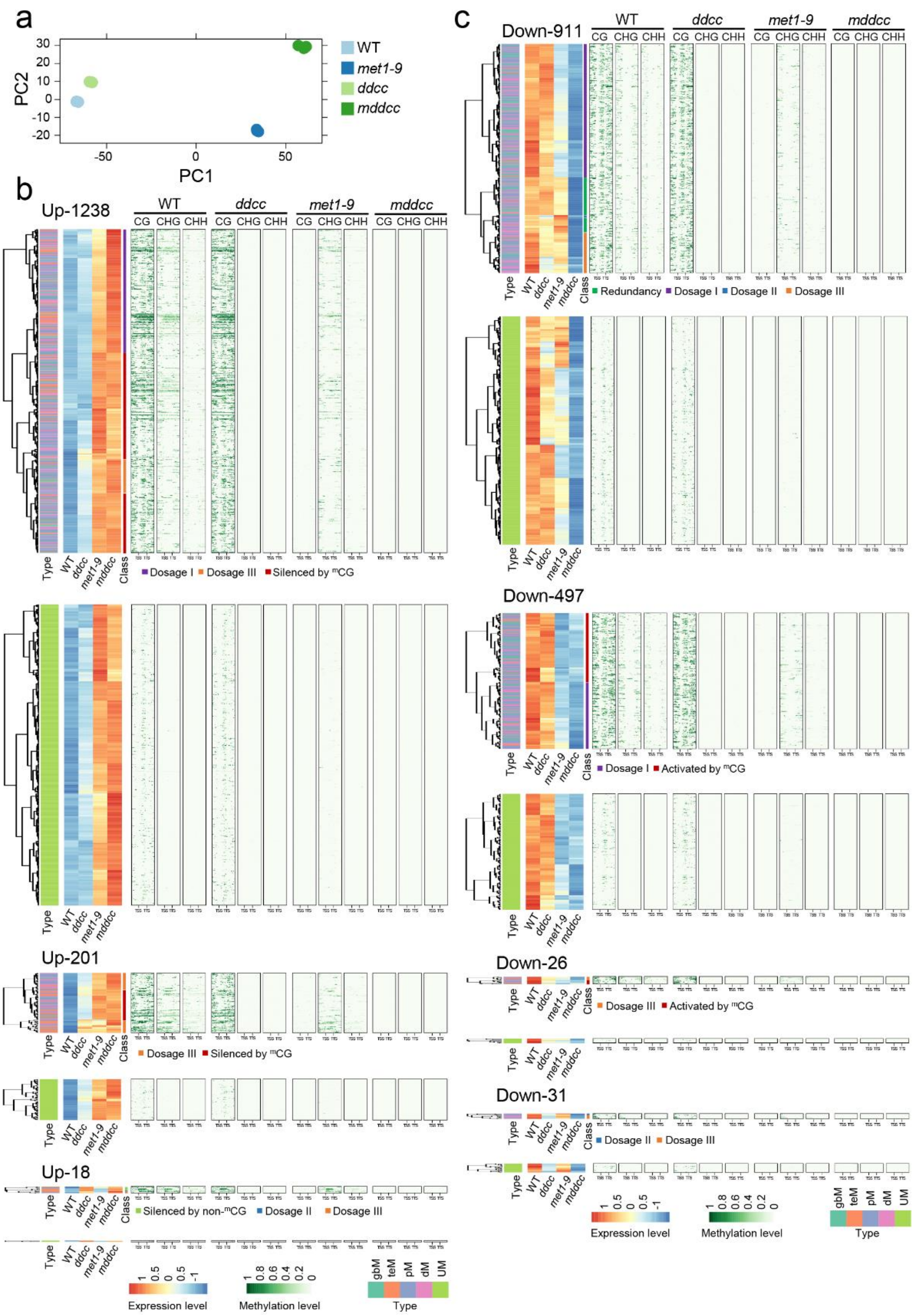

Supplementary Figure 3. DNA methylation regulates gene expression in multiple ways. (a) Principle component analysis of the indicated transcriptomes. (b)-(c) Heat maps showing the expression and DNA methylation patterns of the indicated groups of genes from up-regulated genes (b) or down-regulated genes (c) in DNA methyltransferases-deficient mutants. Selected subsets of genes are taken from those in Figure 2b or 2c. Based on their expression patterns, methylated DEGs were classified into Redundancy, Dosage I, Dosage II, Dosage III, Silenced/Activated by ^{m}CG (the degree of gene up- or down-regulation in *met1-9* is comparable to that in *mddcc*), and Silenced by non-^{m}CG (the degree of gene up-regulation is similar between *ddcc* and *mddcc*); for details, please refer to the main text.

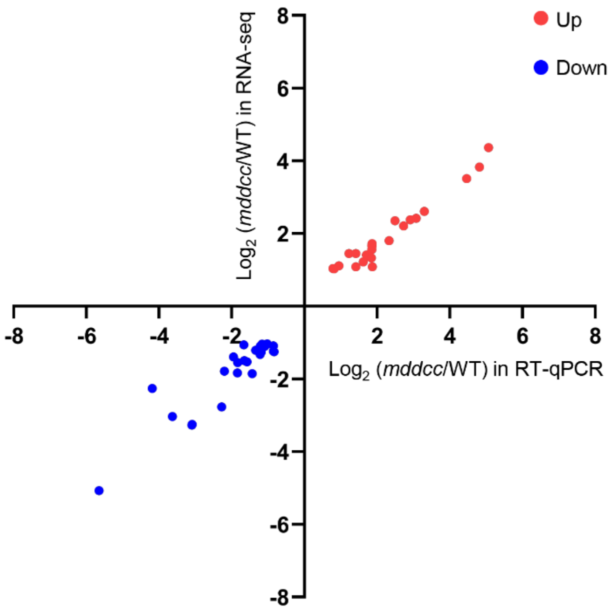

Supplementary Figure 4. Validation of the RNA-seq results by RT-qPCR. x-axis scales represent Log_2 (*mddcc*/WT), measured by RT-qPCR; y-axis scales represent Log_2 (*mddcc*/WT), measured by RNA-seq. Each data point is an average of three biological replicates. Three genes whose expression values are zero either in WT or *mddcc* were excluded in this scatter plot. Source data are provided as a Source Data file.

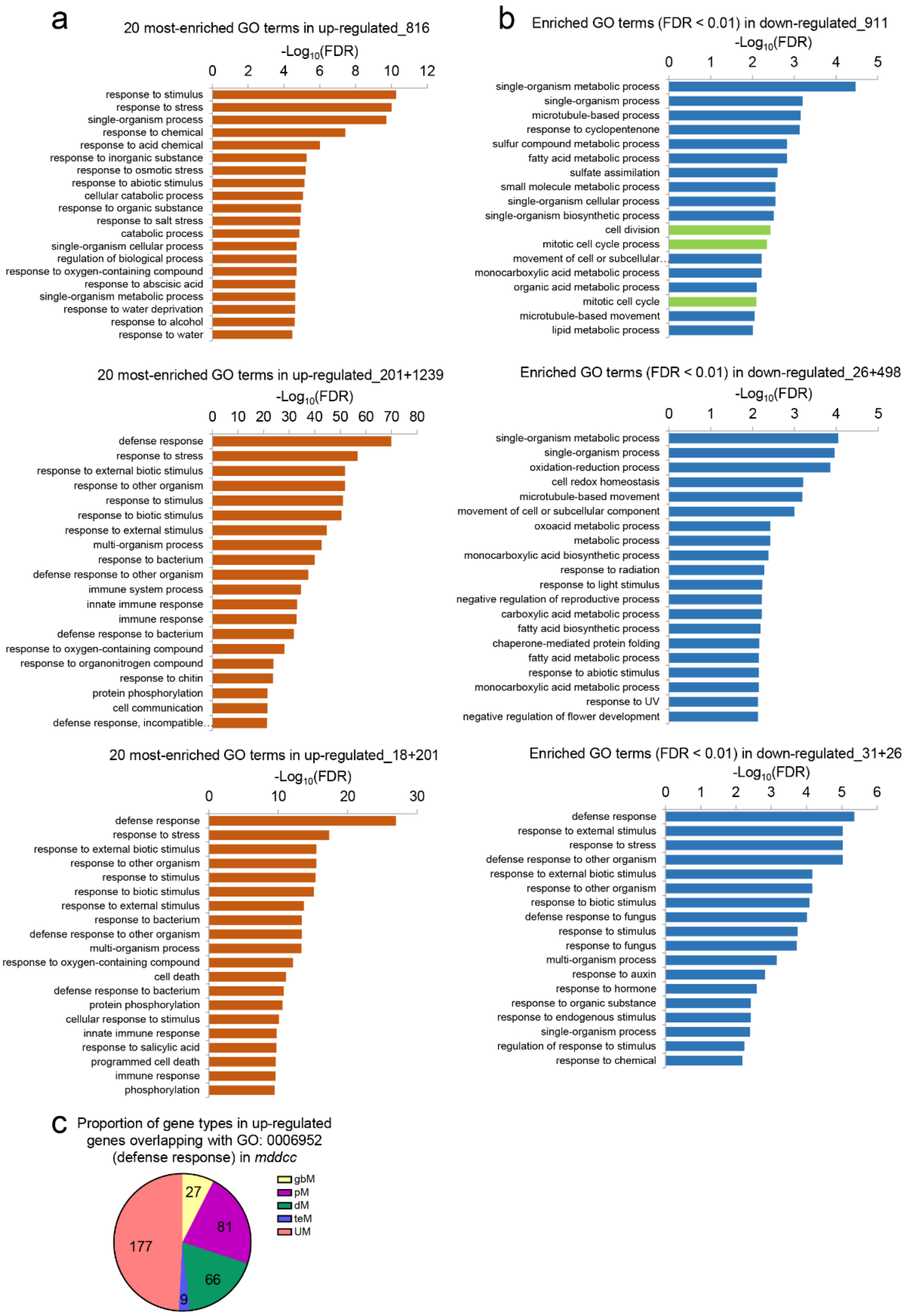

Supplementary Figure 5. GO analysis of DEGs. (a) Functional enrichment analysis of the indicated groups of up-regulated genes (for details, see Figure 2b). Considering that there are many GO terms with FDR < 0.01, only 20 most-enriched GO terms are shown in each case. (b) Functional enrichment analysis of the indicated groups of down-regulated genes (for details, see Figure 2c). GO terms with FDR < 0.01 are shown. Green colors highlight the cell division-related GO terms. (c) Proportion of gene types in the subset of up-regulated genes in *mddcc* within GO:0006952 (defense response).

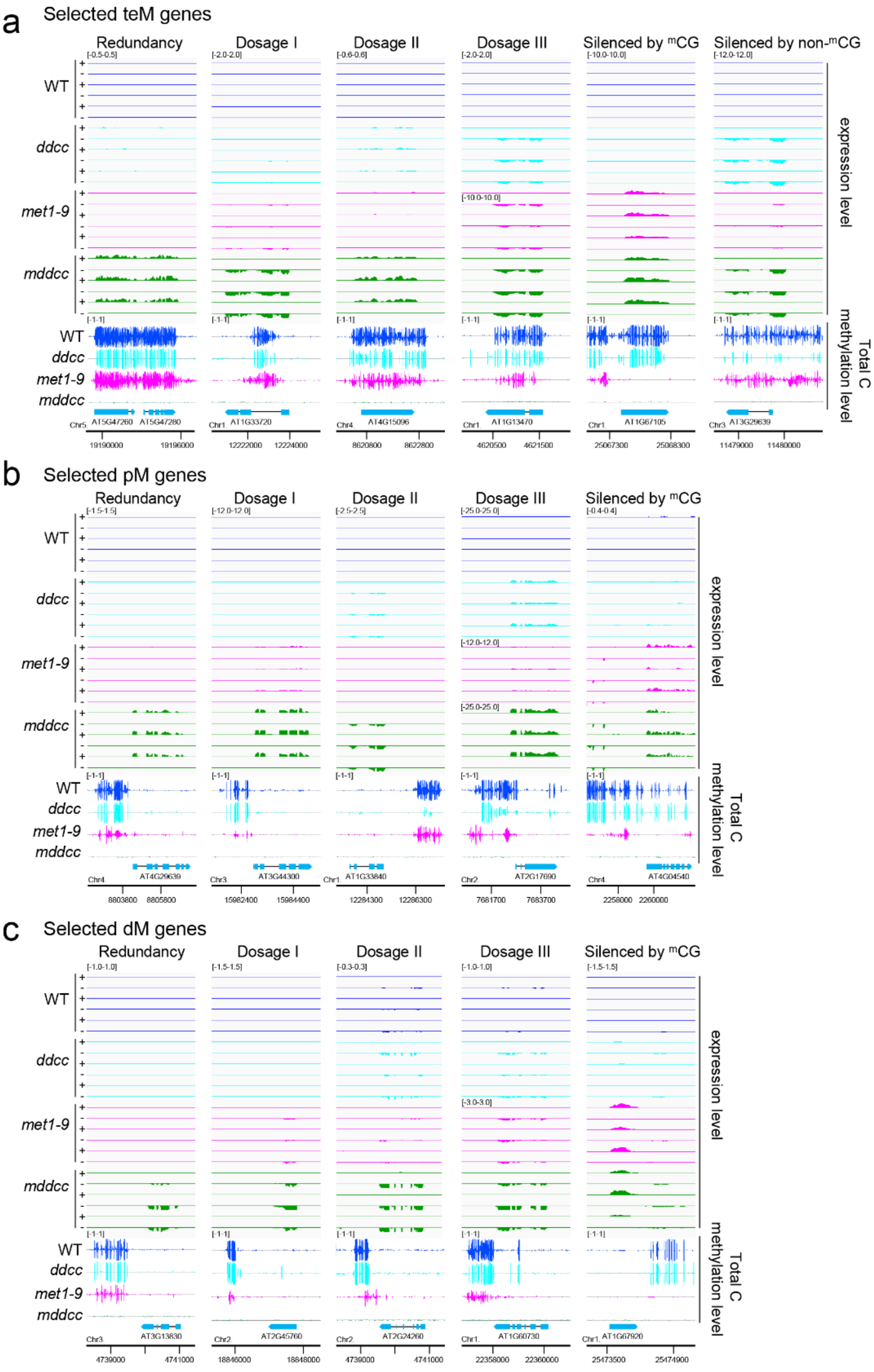

Supplementary Figure 6. Examples of DNA methylation repressing gene expression. (a)-(c) Snapshots of expression and DNA methylation levels over selected genes from the teM (a), pM (b), or dM (c) categories.

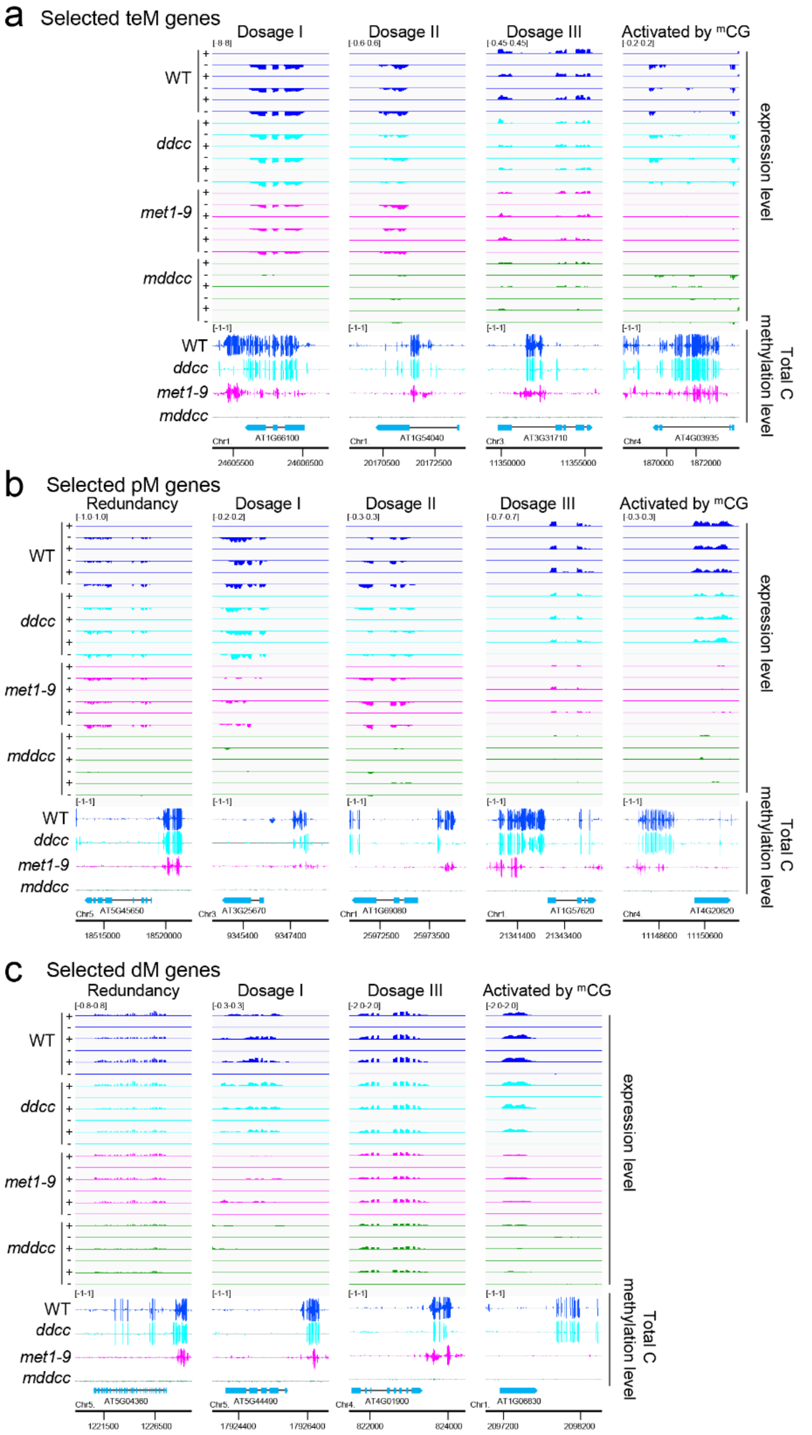

Supplementary Figure 7. Examples of DNA methylation correlating with gene expression. (a)-(c) Snapshots of expression and DNA methylation levels over selected genes from the teM (a), pM (b), or dM (c) categories.

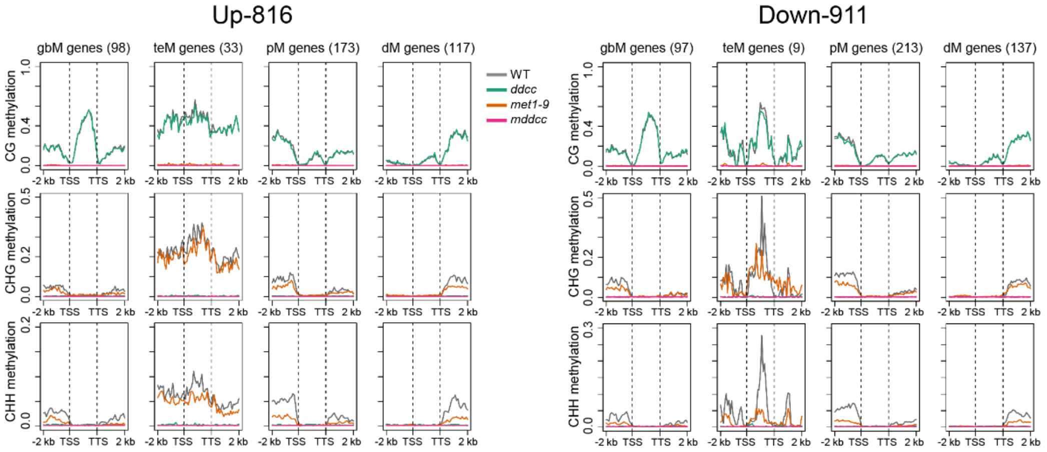

Supplementary Figure 8. Metaplot of CG, CHG, and CHH methylation levels over methylated DEGs. Selected subsets of genes are taken from those in Figure 2b or 2c. The numbers in parentheses indicate the numbers of corresponding gene types.

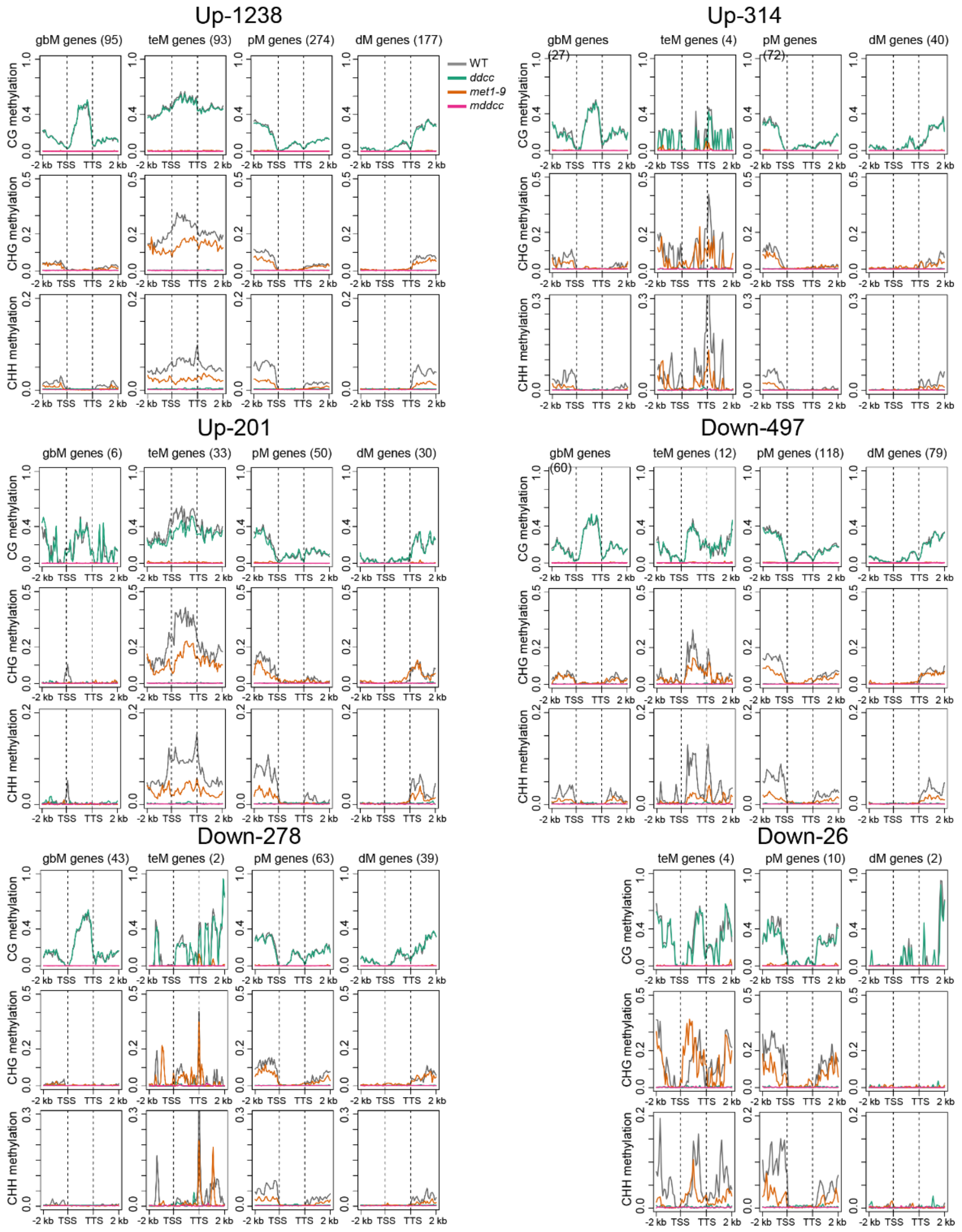

Supplementary Figure 9. Metaplot of CG, CHG, and CHH methylation levels over methylated DEGs from the *met1-9* mutant. Selected subsets of genes are taken from those in Figure 2b or 2c. The numbers in parentheses indicate the numbers of corresponding gene types.

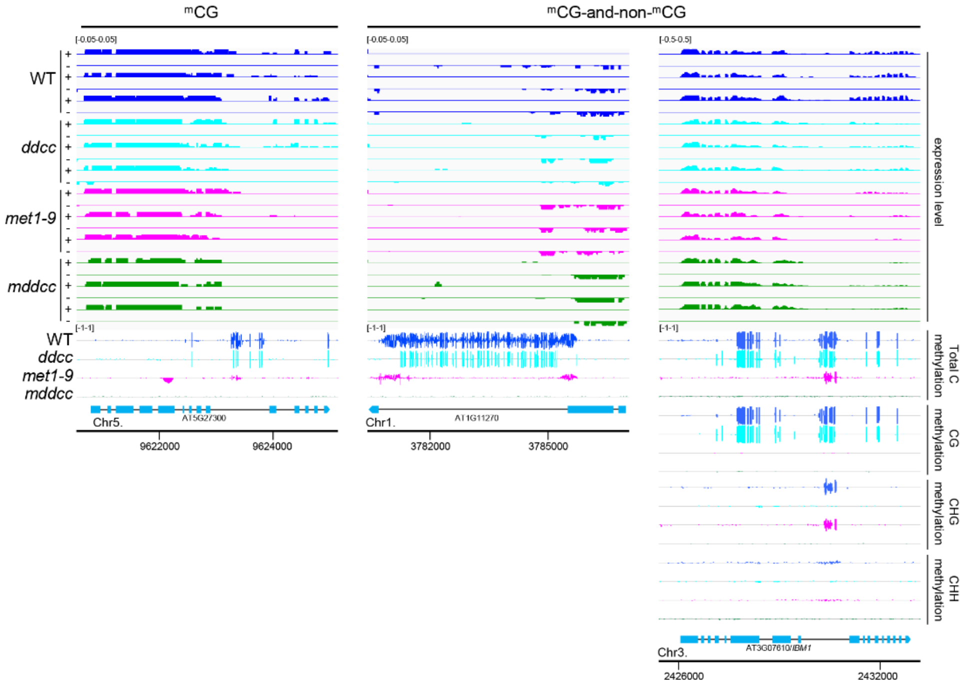

Supplementary Figure 10. Examples of DNA methylation promoting full-length transcripts of Intron-teM genes. Snapshots of expression and DNA methylation levels over selected Intron-teM genes.

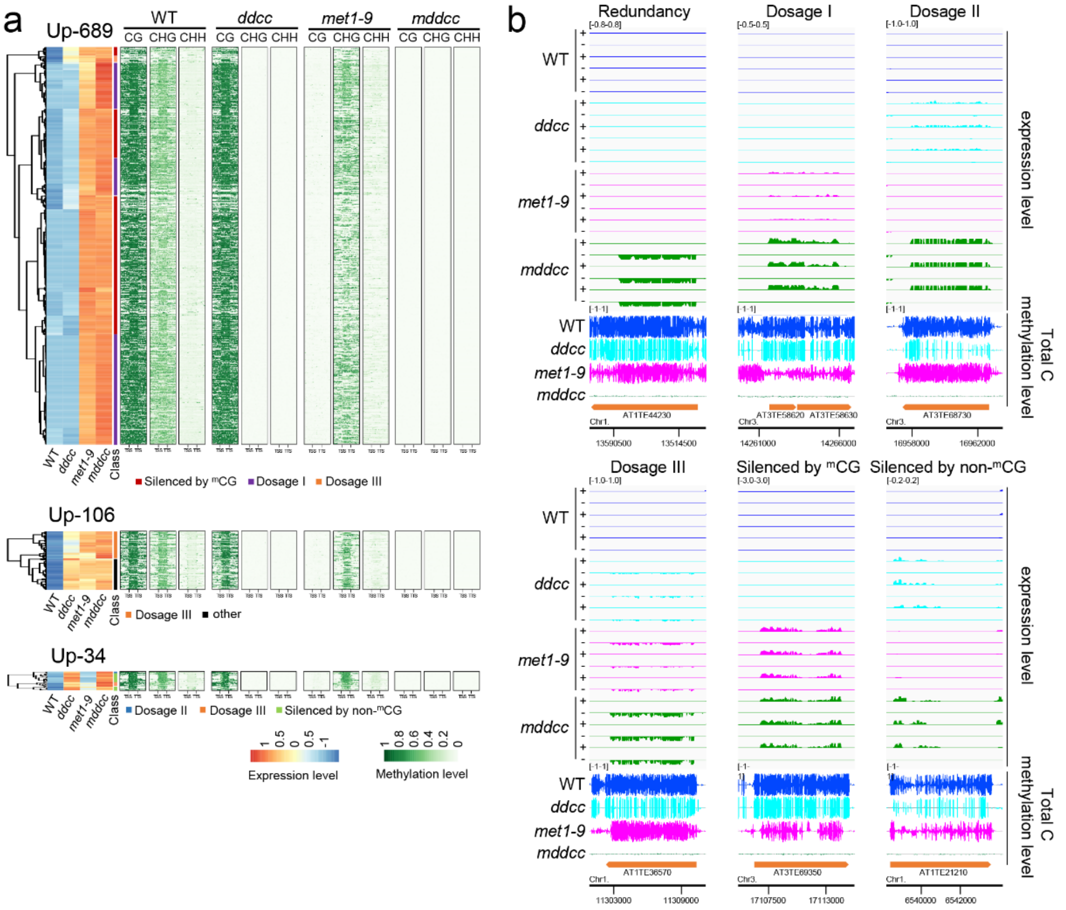

Supplementary Figure 11. DNA methylation represses TEs expression. (a) Heat maps showing the expression and DNA methylation patterns of TEs in the indicated groups (from Figure 3b). Based on their expression pattern, TEs were classified into Dosage I, Dosage II, Dosage III, Silenced by ^{m}CG, Silenced by non-^{m}CG and other (TEs expression levels are similar among *ddcc*, *met1-9* and *mddcc*); for details, please refer to the main text. (b) Examples of DNA methylation repressing TEs expression.

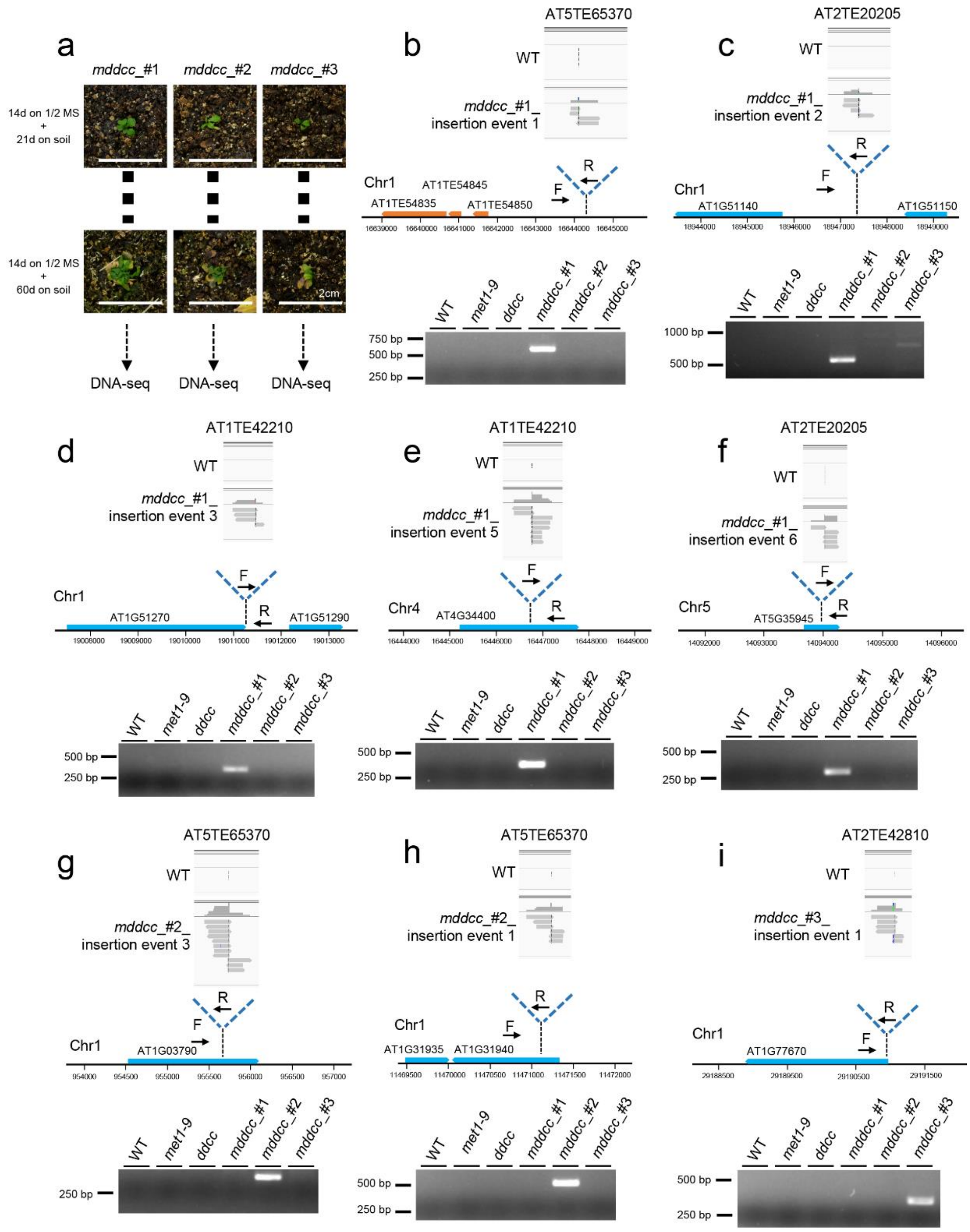

Supplementary Figure 12. Confirmation of TE transpositions by PCR. (a) Images of individual *mddcc* plants used for DNA-seq. (b)-(i) Products of PCR amplification with paired primers flanking the new insertion site or a transposon-specific primer and a primer flanking the new insertion site in the indicated genotypes. Black arrows indicate primers. Upper panels, screenshots from Integrative Genomic Viewer (IGV) showing split-reads for TEs insertion. Middle panels, coordinate lines indicate the sequence contexts of TEs insertion sites. Source data are provided as a Source Data file.

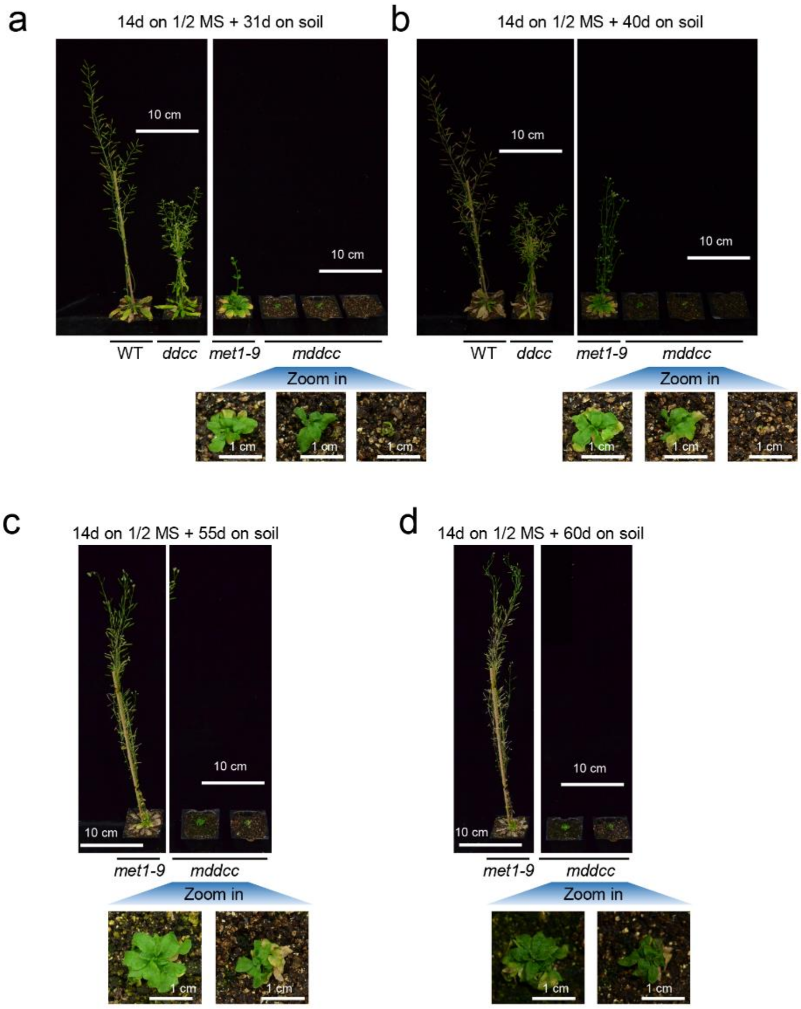

Supplementary Figure 13. Phenotypes of *mddcc* plants in a time-course. (a)-(d) Representative images of *mddcc* plants at different times, as indicated: a, 45-day-old plants; b, 54-day-old plants; c, 69-day-old plants; d, 74-day-old plants. WT, *ddcc*, and *met1-9* plants are shown for comparison.

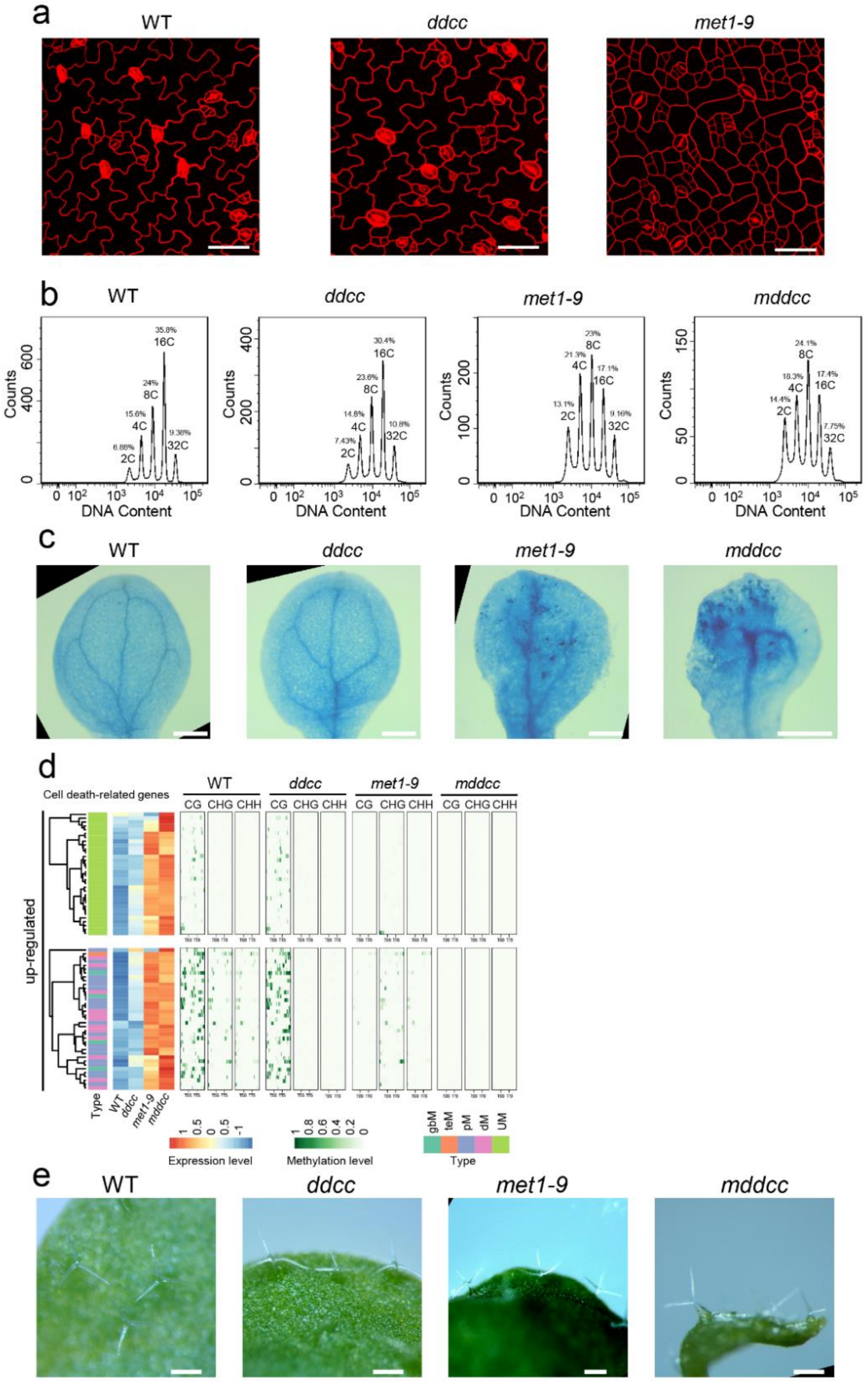

Supplementary Figure 14. DNA methylation regulates pavement cell shape, cell death, endoreduplication, and trichome morphology. (a) Representative images of pavement cell morphology of cotyledons from 4-day-old WT, *ddcc*, and *met1-9* seedlings. Cell outlines were visualized with PI. Scale bar: 50 μm. Experiments were independently repeated two times with similar results. (b) DNA contents determined by flow cytometry using cotyledons from 11-day-old WT, *ddcc*, *met1-9,* and *mddcc* seedlings. (c) Representative images of cotyledons showing cell death detected by trypan blue staining in 11-day-old WT, *ddcc*, *met1-9,* and *mddcc* seedlings. Scale bar: 500 μm. (d) Heat maps showing the expression and DNA methylation patterns of cell death-related genes in the indicated genotypes. Cell death-related genes (see Supplementary Data 1) were identified from the overlap between GO:0008219 (cell death) and up-regulated genes in *mddcc*. (e) Representative images showing trichomes in the first true leaves of 11-day-old WT, *ddcc*, *met1-9,* and *mddcc* seedlings. Scale bar: 200 μm.

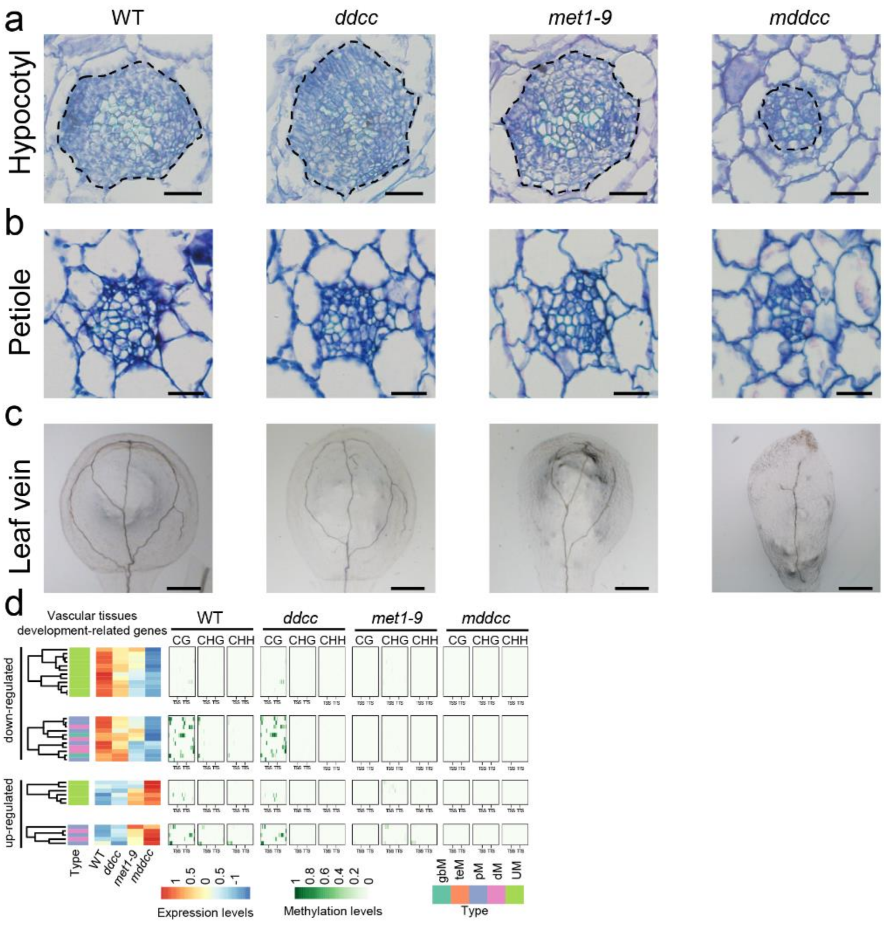

Supplementary Figure 15. CG and non-CG methylation redundantly regulate vascular development. (a) Representative sections across the hypocotyls of 11-day-old WT, *ddcc*, *met1-9,* and *mddcc* seedlings. Scale bar: 25 µm. Experiments were independently repeated one times with similar results. (b) Representative sections across the petioles of cotyledons of 11-day-old WT, *ddcc*, *met1-9,* and *mddcc* seedlings. Scale bar: 20 µm. Experiments were independently repeated one times with similar results. (c) Representative images showing the venation pattern of cotyledons of 11-day-old WT, *ddcc*, *met1-9,* and *mddcc* seedlings. Scale bar: 500 µm. Experiments were independently repeated one times with similar results. (d) Heat maps showing the expression and DNA methylation patterns of vascular development-related genes in the indicated genotypes. Vascular development-related genes were identified from the overlap between DEGs in *mddcc* and GO:0010087 (phloem or xylem histogenesis), GO:0010051 (xylem and phloem pattern formation) or genes references[1] and[2].

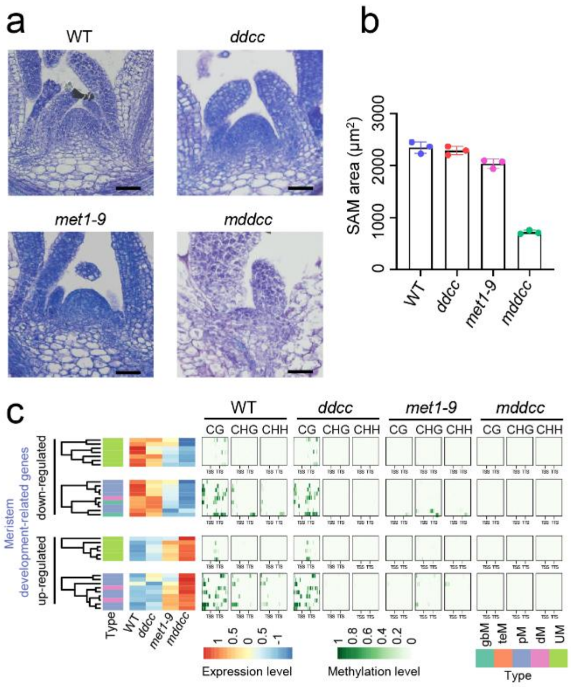

Supplementary Figure 16. DNA methylation regulates SAM size. (a) Representative longitudinal section of the shoot apical meristem of 11-day-old WT, *ddcc*, *met1-9,* and *mddcc* seedlings. Scale bar: 50 μm. Experiments were independently repeated one times with similar results. (b) Quantification of SAM area in the indicated genotypes. The data are the means ± S.D. of the biological repeats (n=3). (c) Heat maps showing the expression and DNA methylation patterns of meristem development-related genes in the indicated genotypes. Meristem development-related genes (see Supplementary Data 1) were identified from the overlap between GO:0048507 (meristem development) and DEGs in *mddcc*. Source data are provided as a Source Data file.

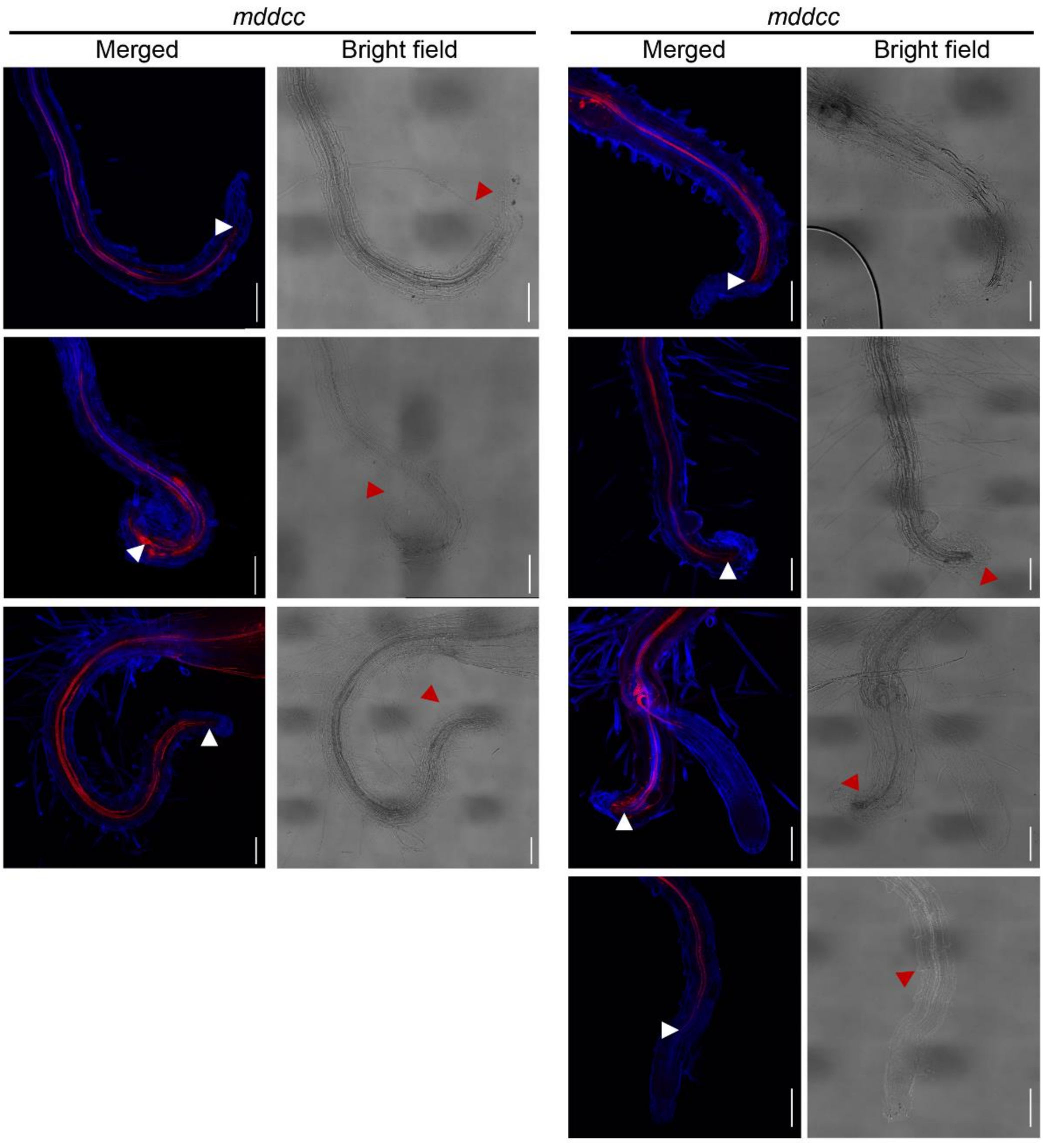

Supplementary Figure 17. Root morphology of 11-day-old *mddcc* seedlings. Representative microscopy images of *mddcc* mutant roots. Cleared roots stained with calcofluor white (blue) and basic fuchsin (red). White arrowheads indicate the site of appearance of the first protoxylem cells; red arrowheads indicate root hairs. Scale bars: 100 μm. Experiments were independently repeated at least two times with similar results.

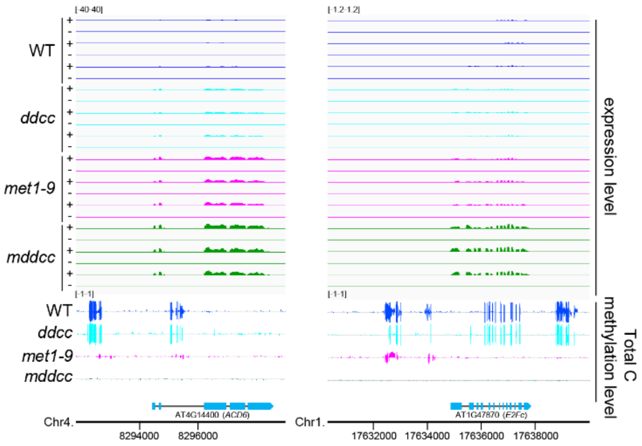

Supplementary Figure 18. Snapshots of expression and DNA methylation levels of *ACD6* and *E2Fc*.

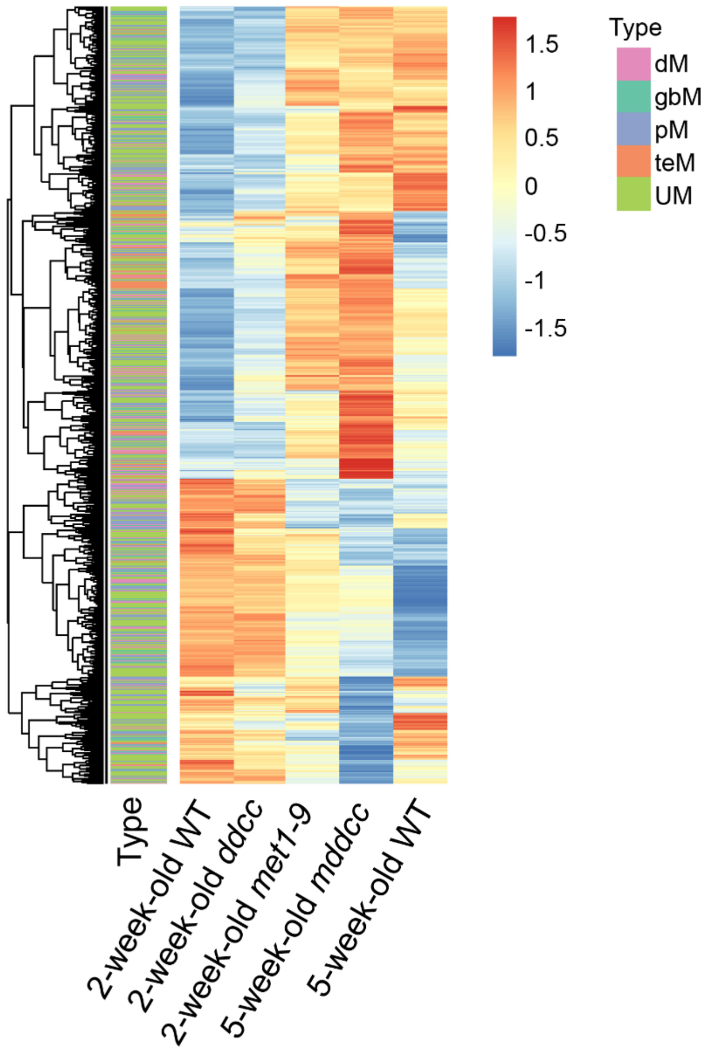

Supplementary Figure 19. Expression profile of DEGs in 5-week-old *mddcc* relative to 2-week-old WT among the indicated genotypes.

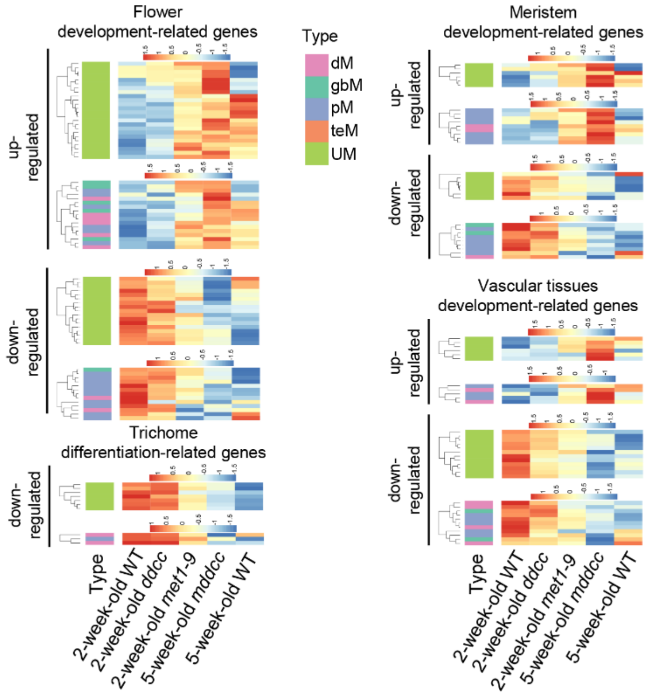

Supplementary Figure 20. Comparing the expression levels of selected DEGs (5-week-old *mddcc* relative to 2-week-old WT) potentially involved in the specific phenotypes of *mddcc* among the indicated genotypes.

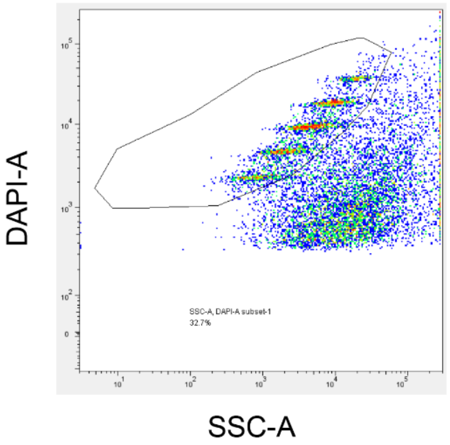

Supplementary Figure 21. Flow cytometry gating strategy for analysis of DNA content.

Supplementary Table 1. Summary of whole-genome bisulfite sequencing results.

**Samples**	**Total reads**	**Clean reads**	**Valid mappings**	**Average coverage**	**Conversion rates**
WT_replicates 1	39139223	34191895	33132845	14.83	99.79%
WT_replicates 2	26649050	24461165	21171418	9.31	99.86%
*ddcc*_replicates 1	34478574	28905441	28928932	12.54	99.74%
*ddcc*_replicates 2	23583553	21249060	18326908	8.1	99.88%
*met1-9*_replicates 1	43989870	43350636	24038413	13.13	99.76%
*met1-9*_replicates 2	53529716	52751089	29652650	16.02	99.78%
*mddcc*_replicates 1	46622456	45803700	39437173	18.12	99.85%
*mddcc*_replicates 2	34406557	34083836	29326757	13.4	99.90%
*mddcc*_replicates 3	32385670	28432763	22134569	8.99	99.90%

Supplementary Table 2. Summary of RNA sequencing results.

**Samples**	**Total reads**	**Clean reads**	**Unique mapped rates**	**Mutiple mapped rates**
WT_replicates 1	33857672	29764885	48.83%	50.97%
WT_replicates 2	34847108	30396787	46.35%	53.41%
WT_replicates 3	30600113	26572700	50.49%	49.27%
*ddcc*_replicates 1	28234524	24440928	50.11%	49.32%
*ddcc*_replicates 2	31972793	27892239	51.88%	47.58%
*ddcc*_replicates 3	32667938	28512310	48.58%	51.17%
*met1-9*_replicates 1	30436432	26565552	49.70%	50.10%
*met1-9*_replicates 2	31145791	27390165	50.56%	49.21%
*met1-9*_replicates 3	27075192	22102626	48.14%	51.56%
*mddcc*_replicates 1	28097660	21153102	50.73%	48.73%
*mddcc*_replicates 2	31447449	23900420	56.29%	43.12%
*mddcc*_replicates 3	27124679	20649099	51.92%	47.27%

Supplementary Table 3. List of 3' down-regulated Intron-teM genes in *ddcc*, *met1-9*, and *mddcc*.

Gene ID	WT 5' reads	WT 3' reads	Log2[(ddcc 3'/5')/(WT 3'/5')]	Log2[(met1-9 3'/5')/(WT 3'/5')]	Log2[(mddcc 3'/5')/(WT 3'/5')]
AT1G58602	201	2379	-0.311878937	-0.801366048	-8.070974471
AT5G27300	111.3333	4	0.093911569	-2.406282332	-4.073740275
AT1G25054	81	3.333333	-4.964805157	-5.03479086	-5.400393985
AT4G28570	77.33333	0	-0.036789797	-1.244229175	-1.809571248
AT3G05410	72	45.66667	-1.300078305	-1.641807502	-7.334874706
AT1G24880	65.66667	3.333333	-1.734164302	-0.595144209	-0.176647494
AT3G46490	37.66667	52	1.035590261	-0.464169917	-3.402769373
AT1G33080	18.66667	15.33333	-0.331221573	-0.99798519	-1.346894952
AT3G28100	16.33333	39	-0.610294565	-0.990758652	-1.002882606
AT1G11270	10	2	-3.950043339	-4.188062429	-4.940306328

Supplementary Table 4. Summary of DNA sequencing results.

Samples	Raw reads	Clean reads	Multiple mapped rates	Unique mapped rates
WT/#1	25137503	23490330	79%	51%
ddcc	28316030	26385302	78%	51%
met1-9	209285<br>vcv48	19984580	84.20%	58.30%
*mddcc*_#1	23077648	21700523	41.30%	26.50%
*mddcc*_#2	26793756	25102160	42.60%	27.20%
*mddcc*_#3	30479327	28629913	24.40%	15.70%

Supplementary Table 5. Summary of TE transposition events identified in *mddcc.*

Samples	Insertion events	Chromosome	Start	End	TE	Family	Validation of PCR
ddcc	1	5	10532403	10532418	AT1TE38795	DNA/MuDR	FALSE
met1-9	1	1	13402134	13402153	AT2TE20085	LTR/Gypsy	FALSE
*mddcc*_#1	1	1	16644301	16644308	AT5TE65370	LTR/Copia	TRUE
	2	1	18947368	18947374	AT2TE20205	DNA/En-Spm	TRUE
	3	1	19011273	19011280	AT1TE42210	DNA/En-Spm	TRUE
	4	3	2999842	2999846	AT1TE42210	DNA/En-Spm	TRUE
	5	4	16446738	16446742	AT1TE42210	DNA/En-Spm	TRUE
	6	5	14093978	14093981	AT2TE20205	DNA/En-Spm	TRUE
*mddcc*_#2	1	1	11471129	11471136	AT5TE65370	LTR/Copia	TRUE
	2	1	15202289	15202294	AT1TE49860	DNA	FALSE
	3	1	955661	955667	AT5TE65370	LTR/Copia	TRUE
	4	2	14021144	14021155	AT2TE42810	DNA/MuDR	TRUE
*mddcc*_#3	1	1	29190880	29190890	AT2TE42810	DNA/MuDR	TRUE
	2	3	4120598	4120603	AT2TE20205	DNA/En-Spm	TRUE

Supplementary references

1 Ruonala, R., Ko, D. & Helariutta, Y. Genetic Networks in Plant Vascular Development. *Annu. Rev. Genet.* **51**, 335-359 (2017).

2 Cano-Delgado, A., Lee, J. Y. & Demura, T. Regulatory mechanisms for specification and patterning of plant vascular tissues. *Annu. Rev. Cell Dev. Biol.* **26**, 605-637 (2010).
