# Supplementary material for: DNA methylation-free Arabidopsis reveals crucial roles of DNA methylation in regulating gene expression and development
Source: Nat Commun. 2022 Mar 14;13:1335. doi: 10.1038/s41467-022-28940-2 (PMC8921224; doi:10.1038/s41467-022-28940-2)
Supplement: Supplementary file 3 — Description of Additional Supplementary Files [file 41467_2022_28940_MOESM3_ESM.pdf]

Description of Additional Supplementary Files

File Name: Supplementary Data 1
Description: List of DEGs in mddcc involved in indicated functional categories

File Name: Supplementary Data 2
Description: Primers used in this study

File Name: Supplementary Data 3
Description: Definition of DNA methylation-associated genes
