# Supplementary material for: DNA methylation-free Arabidopsis reveals crucial roles of DNA methylation in regulating gene expression and development
Source: Nat Commun. 2022 Mar 14;13:1335. doi: 10.1038/s41467-022-28940-2 (PMC8921224; doi:10.1038/s41467-022-28940-2)
Supplement: Supplementary file 7 — Reporting Summary [file 41467_2022_28940_MOESM7_ESM.pdf]

nature portfolio

Corresponding author(s): Jian-Kang Zhu

Last updated by author(s): Jan 16, 2022

Reporting Summary

Nature Portfolio wishes to improve the reproducibility of the work that we publish. This form provides structure for consistency and transparency in reporting. For further information on Nature Portfolio policies, see our Editorial Policies and the Editorial Policy Checklist.

Statistics

For all statistical analyses, confirm that the following items are present in the figure legend, table legend, main text, or Methods section.

n/a	Confirmed	
☐	☒	The exact sample size (*n*) for each experimental group/condition, given as a discrete number and unit of measurement
☐	☒	A statement on whether measurements were taken from distinct samples or whether the same sample was measured repeatedly
☐	☒	The statistical test(s) used AND whether they are one- or two-sided<br>*Only common tests should be described solely by name; describe more complex techniques in the Methods section.*
☒	☐	A description of all covariates tested
☐	☒	A description of any assumptions or corrections, such as tests of normality and adjustment for multiple comparisons
☐	☒	A full description of the statistical parameters including central tendency (e.g. means) or other basic estimates (e.g. regression coefficient) AND variation (e.g. standard deviation) or associated estimates of uncertainty (e.g. confidence intervals)
☐	☒	For null hypothesis testing, the test statistic (e.g. *F*, *t*, *r*) with confidence intervals, effect sizes, degrees of freedom and *P* value noted<br>*Give P values as exact values whenever suitable.*
☒	☐	For Bayesian analysis, information on the choice of priors and Markov chain Monte Carlo settings
☒	☐	For hierarchical and complex designs, identification of the appropriate level for tests and full reporting of outcomes
☒	☐	Estimates of effect sizes (e.g. Cohen's *d*, Pearson's *r*), indicating how they were calculated

Our web collection on statistics for biologists contains articles on many of the points above.

Software and code

Policy information about availability of computer code

Data collection	HiSeq X Ten; BD FASCAria III flow cytometer
Data analysis	BS-seq and RNA-seq analysis: Trimmomatic version 0.36, Cutadapt version 1.16, BSMAP version 2.9.0, Bowtie2 version 2.3.4.1, FastQC version v0.11.7, HTSeq version 0.9.1, Cufflink version v2.2.1, DESeq2 version 1.14.1, featureCounts version 1.14.1, STAR version v2.5.3<br>DNA-seq analysis: Bowtie2 version 2.2.5, SPLITREADER beta1.1<br>Data visualization: Integrative Genomics Viewer version 2.1.24, Integrated Genome Browser version 9.0.1<br>GO analysis: agriGO version 2<br>FACS data analysis: FlowJo 7.6

For manuscripts utilizing custom algorithms or software that are central to the research but not yet described in published literature, software must be made available to editors and reviewers. We strongly encourage code deposition in a community repository (e.g. GitHub). See the Nature Portfolio guidelines for submitting code & software for further information.

Data

Policy information about availability of data

All manuscripts must include a data availability statement. This statement should provide the following information, where applicable:

- Accession codes, unique identifiers, or web links for publicly available datasets
- A description of any restrictions on data availability
- For clinical datasets or third party data, please ensure that the statement adheres to our policy

All high-throughput sequencing data generated in this study have been deposited in GEO with accessions codes GSE169497. All data supporting the findings of this study are available within the manuscript and its supplementary files. Source data are provided with this paper.

Field-specific reporting

Please select the one below that is the best fit for your research. If you are not sure, read the appropriate sections before making your selection.

- [x] Life sciences
- [ ] Behavioural & social sciences
- [ ] Ecological, evolutionary & environmental sciences

For a reference copy of the document with all sections, see nature.com/documents/nr-reporting-summary-flat.pdf

Life sciences study design

All studies must disclose on these points even when the disclosure is negative.

Sample size	Sample size in all the experiments was sufficient for statistical significance and reproducibility. For BS-seq and RNA-seq, at least 6 seedlings were collected for each sample. For the analysis of phenotypes, at least 3 plants (ranged from 3 to 49 plants for different experiments) were used for the measurement.
Data exclusions	No data were excluded from the analysis.
Replication	RNA-seq was performed with three biological replicates. BS-seq was performed with two biological replicates (with the exception of mddcc mutant, for which three replicates were used). DNA-seq was performed with one biological replicate (with the exception of mddcc mutant, for which three replicates were used). All attempts at replication were successful.
Randomization	Arabidopsis plants grown under same conditions were randomly selected.
Blinding	No blinding was used. When processing the sequencing data, the same parameters were applied. There was no possible manual intervention.

Reporting for specific materials, systems and methods

We require information from authors about some types of materials, experimental systems and methods used in many studies. Here, indicate whether each material, system or method listed is relevant to your study. If you are not sure if a list item applies to your research, read the appropriate section before selecting a response.

Materials & experimental systems

n/a	Involved in the study	
x		Antibodies
x		Eukaryotic cell lines
x		Palaeontology and archaeology
x		Animals and other organisms
x		Human research participants
x		Clinical data
x		Dual use research of concern

Methods

n/a	Involved in the study	
x		ChIP-seq
	x	Flow cytometry
x		MRI-based neuroimaging

Flow Cytometry

Plots

Confirm that:

- [x] The axis labels state the marker and fluorochrome used (e.g. CD4-FITC).
- [x] The axis scales are clearly visible. Include numbers along axes only for bottom left plot of group (a 'group' is an analysis of identical markers).
- [x] All plots are contour plots with outliers or pseudocolor plots.
- [x] A numerical value for number of cells or percentage (with statistics) is provided.

Methodology

Sample preparation	Cotyledons of 11-day-old plants were chopped with a razor blade in 1ml Galbraith's buffer (45 mM MgCl2, 30 mM sodium citrate, 20 mM MOPS, 0.1% (v/v) Triton x-100, pH=7.0). The lysate was filtered through a 40-µm cell strainer (BD Falcon) and incubated at 4°C for 5 min. The eluate was transferred to a 15 ml tube, 2 µl DAPI solution (1 mg/ml) were added, and the mixture was incubated on ice for 10 min.
Instrument	BD FASCAria III
Software	FlowJo 7.6

Cell population abundance	At least 1500 cells were analysed in each genotype.
Gating strategy	Nuclei were isolated from cotyledons of 11-day-old plants and stained by propidium iodide. The relative fluorescence intensity indicated DNA content and ploidy of the nuclei. FACS was used to sort nuclei with different DNA content.

☒ Tick this box to confirm that a figure exemplifying the gating strategy is provided in the Supplementary Information.
